# Transcriptome Analysis Identifies Tumor Immune Microenvironment Signaling Networks Supporting Metastatic Castration-Resistant Prostate Cancer

**DOI:** 10.3390/onco3020007

**Published:** 2023-04-10

**Authors:** Lawrence P. McKinney, Rajesh Singh, I. King Jordan, Sooryanarayana Varambally, Eric B. Dammer, James W. Lillard

**Affiliations:** 1 Department of Microbiology, Biochemistry, and Immunology, Morehouse School of Medicine, Atlanta, GA 30310, USA; 2 School of Biological Sciences, Georgia Institute of Technology, Atlanta, GA 30332, USA; 3 Division of Molecular and Cellular Pathology, Department of Pathology, The University of Alabama at Birmingham, Birmingham, AL 35233, USA; 4 Department of Biochemistry Emory, University School of Medicine, Atlanta, GA 30329, USA

**Keywords:** transcriptomics, metastasis, tumor microenvironment, prostate cancer, signaling

## Abstract

Prostate cancer (PCa) is the second most common cause of cancer death in American men. Metastatic castration-resistant prostate cancer (mCRPC) is the most lethal form of PCa and preferentially metastasizes to the bones through incompletely understood molecular mechanisms. Herein, we processed RNA sequencing data from patients with mCRPC (*n* = 60) and identified 14 gene clusters (modules) highly correlated with mCRPC bone metastasis. We used a novel combination of weighted gene co-expression network analysis (WGCNA) and upstream regulator and gene ontology analyses of clinically annotated transcriptomes to identify the genes. The cyan module (M14) had the strongest positive correlation (0.81, *p* = 4 × 10^−15^) with mCRPC bone metastasis. It was associated with two significant biological pathways through KEGG enrichment analysis (parathyroid hormone synthesis, secretion, and action and protein digestion and absorption). In particular, we identified 10 hub genes (*ALPL*, *PHEX*, *RUNX2*, *ENPP1*, *PHOSPHO1*, *PTH1R*, *COL11A1*, *COL24A1*, *COL22A1*, and *COL13A1*) using cytoHubba of Cytoscape. We also found high gene expression for collagen formation, degradation, absorption, cell-signaling peptides, and bone regulation processes through Gene Ontology (GO) enrichment analysis.

## Simple Summary

Metastatic castration-resistant prostate cancer (mCRPC) is potentially lethal and often spreads to the bones through a biological mechanism we do not completely understand. A previous study sequenced RNA from patients with mCRPC, and in this study, we have identified 10 genes associated with mCRPC that spread to the bones; two of those genes are novel discoveries that could serve as new biomarkers for diagnosis or molecular targets for treatment. However, future studies are required to validate these genes’ molecular role in mCRPC progression.

## 1. Introduction

The 5-year relative survival rate for prostate cancer (PCa) decreases from >99% to 31% once the disease has metastasized to distant sites [1]. The bones are the most common site for disease spread for metastatic castration-resistant prostate cancer (mCRPC), suggesting that the bone microenvironment is conducive to mCRPC growth and survival.

To date, the molecular mechanisms of mCRPC remain incompletely described. Many studies have examined the clinical characteristics of mCRPC, especially in terms of genetic alterations and the role of the tumor immune microenvironment (TIME) in dynamic tumor evolution. Still, few have fully characterized the mCRPC transcriptome [2–5].

This study captures a global picture of mCRPC cellular function and TIME-related signaling networks in bone tissue. We analyzed mRNA expression data from the Database of Genotypes and Phenotypes (dbGAP) using WGCNA. We critically assessed the relevance of genes differentially expressed in mCRPC by matching clinical annotations with biological gene functions that positively correlate with bone mCRPC through functional enrichment, pathway, and protein–protein interaction (PPI) analyses. These results further elucidate the biological pathways, molecular functions, and clinical events that underpin mCRPC cell migration, survival, and bone metastasis.

## 2. Materials and Methods

### 2.1. Patient Samples and Quality Control Measures

We retrieved the raw RNA-Seq data and matched clinical annotations for 150 mCRPC samples from the 2019 Metastatic Prostate Adenocarcinoma-Standup2Cancer/Prostate Cancer Foundation Dream Team: Precision Therapy for Advanced Prostate Cancer study (dbGaP Study Accession: phs000915.v2.p2). We used the SRA toolkit version 2.9.6.1 to download data with controlled access from the Cbioportal [6] and GitHub [7].

We performed a quality control assessment on all raw sequence reads ahead of alignment to the human reference genome using Fastp v0.20.1 [8]. We removed bad-quality base pairs and contaminated adaptors from the dataset and then used Fastp to check for the presence or absence of overrepresented sequences, the guanine and cytosine (GC) percent distribution, and the proportion of GC base pairs across all reads. Fastp scores the overall sequence quality and overrepresentation to diagnose potential sequence quality issues.

We then mapped 269 patient transcriptomes to the human reference genome (GRCh.38.p13) using STAR v2.7.3a [9]. We performed a quality control assessment on read alignment using FASTQC v0.11.9 by visualization with MULTIQC v1.11 [10]. Low-quality samples, samples possessing no matched clinical data, and RNA-Seq samples not prepared using a hybrid selection or capture method of enrichment were filtered out and removed. Samples prepared using poly-A selection were filtered out because this method would not detect non-coding RNAs such as miRNAs and some lncRNAs needed for future analysis; 198 samples remained.

We then filtered for duplicates/replicates to preserve the integrity of analysis. This reduced our sample size, and 89 samples remained. Samples were originally sequenced using Illumina HiSeq 2000. Years later, samples were re-sequenced using Illumina HiSeq 2500. For consistency, we filtered out the 20 samples that were sequenced using only Illumina HiSeq2000 and proceeded with the samples that were sequenced using Illumina HiSeq2500; 69 samples remained. Finally, we filtered the remaining samples for tissue sites that had *n* < 10, as we needed at least 10 samples in each group to detect differences by tissue site in our analysis. This resulted in our team proceeding with three mCRPC tissue types (liver, lymph node, and bone).

After applying quality control and normalization measures featured in the DESeq2 package [11], we retained 60 mCRPC samples for analyzing RNA-Seq data and clinical characteristics (Table 1). The patients’ median age was 65, and Gleason scores (GS) ranged from 6–10, with 24 samples labeled as unknown (UNK). Four patients had a UNK hormone therapy status. Less than half of the samples (*n* = 25) were exposed to abiraterone and enzalutamide, whereas the remaining patients (*n* = 31) were treatment-naïve. The majority of patients (*n* = 37) were treatment-naïve for taxanes. For our final study total, we only included patient samples retrieved from single metastatic tissue sites in the bone (*n* = 15), lymph node (*n* = 34), or liver (*n* = 11). Other metastatic sites were excluded due to there being so few cases.

### 2.2. Normalization and Gene Expression Quantification

We used featureCounts v1.5.0-p3 to quantify counts from the RNA-Seq data and imported that data in R Studio version 3.6.3 [12]. We normalized read counts with respect to library size using DESeq2 [11] and applied log2 scale transformations to minimize differences between sample rows with <10 counts.

### 2.3. Co-Expression Network Analysis and Module Identification

We transformed the data frame to match the WGCNA format, with samples arrayed across rows and genes across columns. We found no gene or sample outliers that did not pass the criteria on the maximum number of 50% or less missing or low weight values when executing the function goodSamplesGenes(). We then uploaded gene expression and clinical data as a matrix data table, with read count data across rows and clinical annotations across columns. We clustered samples by expression level using signed network connectivity with default parameters. We then produced a heatmap and dendrogram of the sample clusters and clinical traits to visually identify any sample outliers, one of which we removed (sampleID: SRR8311618). We segmented the resultant dataset into three matrices that discerned gene clusters by tissue site (bone, liver, or lymph nodes). In each matrix, we coded samples with a “1” if they were positive for the respective tissue site, whereas all others were assigned a “0”.

**Figure 1.**
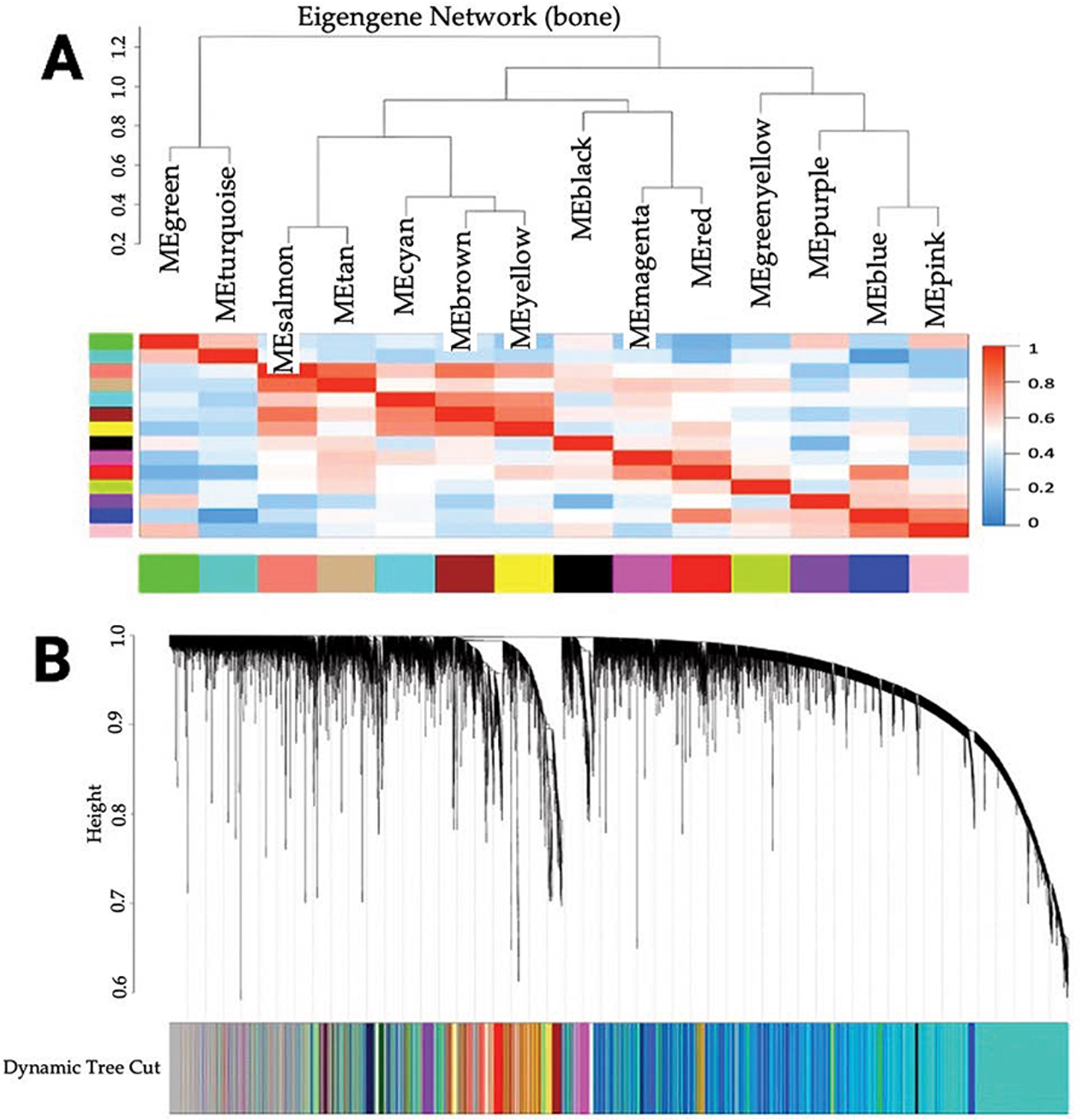
Bone eigengene network/cluster dendrogram. (**A**) Eigengene network and heatmap of clustered dissimilarity based on consensus topological overlap (14 modules) and eigengene heatmap. (**B**) Based on consensus topological overlap, a cluster dendrogram of clustered dissimilarity and module colors.

We constructed a matrix to correlate tissue sites with co-expression amongst genes in the network. We used a manual block-wise network to facilitate WGCNA and perform module detection. We chose an appropriate soft threshold power of β for the network topology analysis based on the scale-free topology criterion [13]; we chose the lowest β that crossed the R^2^ cut-off of 0.90 to yield an approximately scale-free topology, as measured by the scale-free topology fitting index. For the analysis type, we used a signed network adjacency calculation to translate the adjacency into a topological overlap matrix (TOM) and calculated the corresponding dissimilarity as dissTOM = 1-TOM [14]. We generated a cluster tree based on TOM dissimilarity and controlled the minimum number of genes clustered in a module by setting minModuleSize/minClusterSize to 30 and deepSplit to 4, imbuing high sensitivity to cluster splitting. We finally used the Dynamic Cut Tree method to show the eigengene network heatmap and gene cluster dendrogram tree for the 14 eigengene clusters (Figures 1A and 1B, respectively).

**Figure 2.**
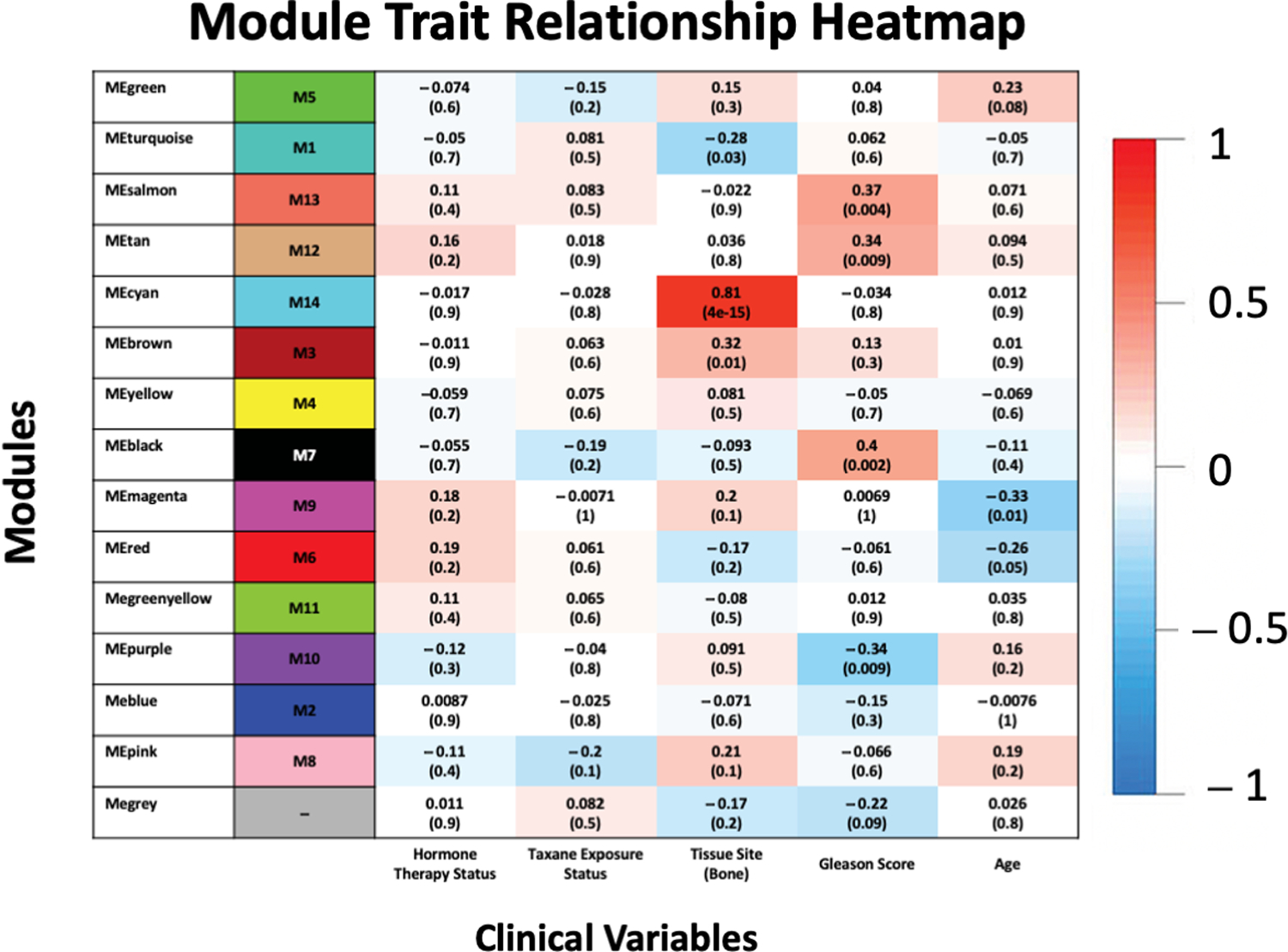
Module trait relationship. Listed in the heatmap are Pearson rho correlations and *p*-values (in parentheses) defining the relationship between ME expression and clinical traits. Each row in the table corresponds to a module with the color shown on the left, and each column corresponds to a specific clinical trait.

We calculated module eigengenes (MEs) based on a vector of color assignments with the same length as the number of gene rows in the data frame. The calculation is the first principal component of the variance for all members of each respective module. We then ranked modules by the number of genes in each module (i.e., the size). We used the function signedkME to reassign membership to the MEs based on connectivity and the Pearson correlation function to calculate the ME co-expression similarity. We saved the resultant consensus kME (eigengene-based connectivity) values and created a heatmap for each clinical trait using the first principal component of each module (Figure 2). These MEs were representatives of all genes in each module [14,15].

### 2.4. Enrichment Analysis, Differential Expression Analysis, and Hub Gene Identification

We used functional annotation approaches to explore the biological function of ME genes, including Gene Ontology (GO) and Kyoto Encyclopedia of Genes and Genomes (KEGG) pathway analyses. We conducted a pathway enrichment analysis for genes co-expressed in the cyan module (Module 14, M14), which had a high corr222-elation with bone tissues metastases, using the Database for Annotation, Visualization, and Integrated Discovery (DAVID version 6.8) [16]. The cyan (M14) module contained 37 genes, from which we identified the top three highly enriched GO terms for each GO subdomain and KEGG pathway. In Table 2, we outline a list of significant genes and signaling pathway terms from the KEGG pathway analysis.

**Figure 3.**
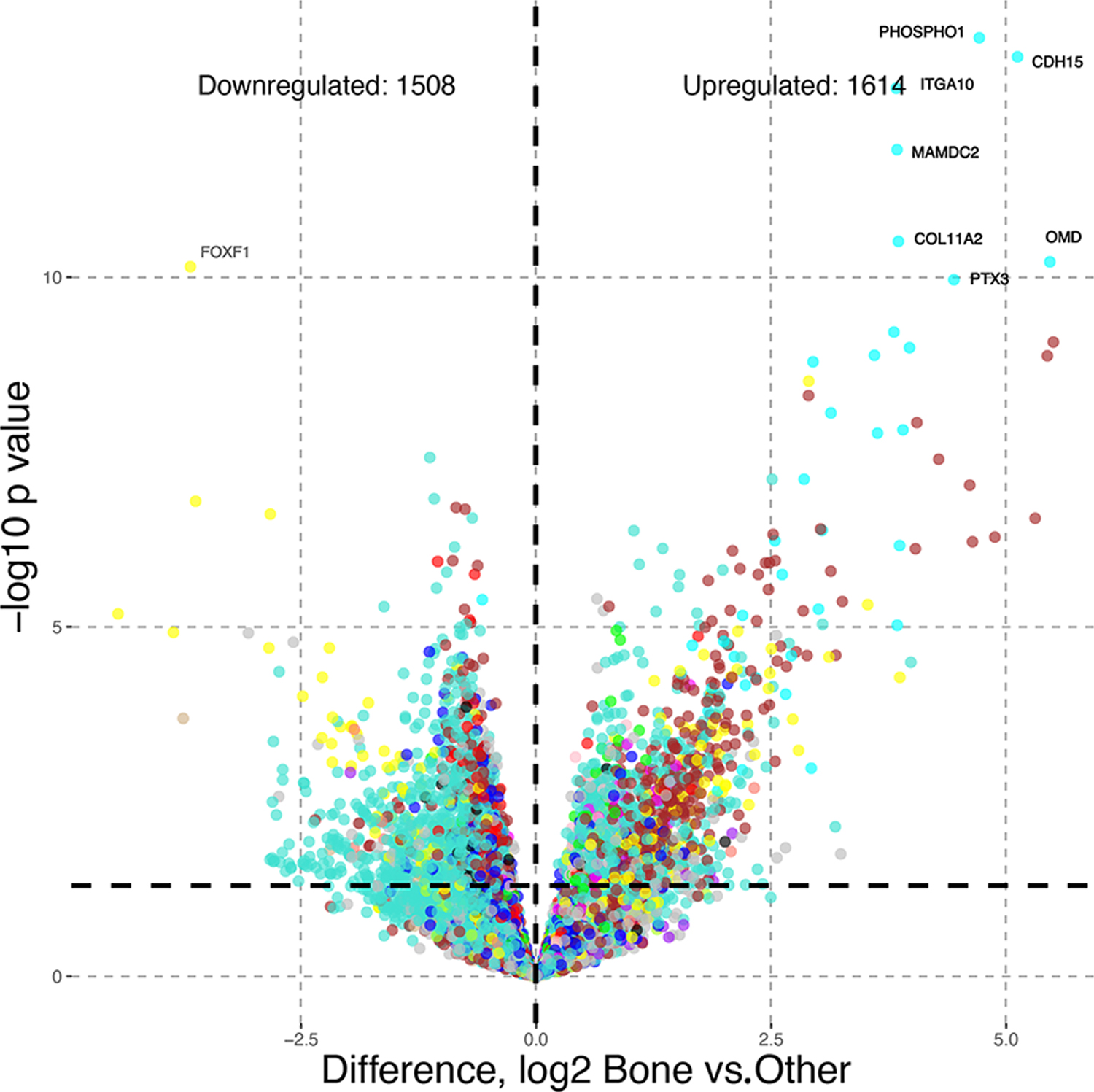
Differential gene expression analysis identified 3122 genes that were upregulated or downregulated when comparing mCRPC samples with bone metastases to samples with other metastatic tissue sites (lymph nodes and liver). Differentially expressed genes with *p* < 1 × 10^−10^ are labeled.

We adapted a method for differential gene expression analysis [5] to identify upregulated or downregulated genes in the MEs and to analyze differences between the metastatic sites. For example, Figure 3 shows differential gene expression for bone metastases versus other metastatic sites (lymph node and liver).

**Figure 4.**
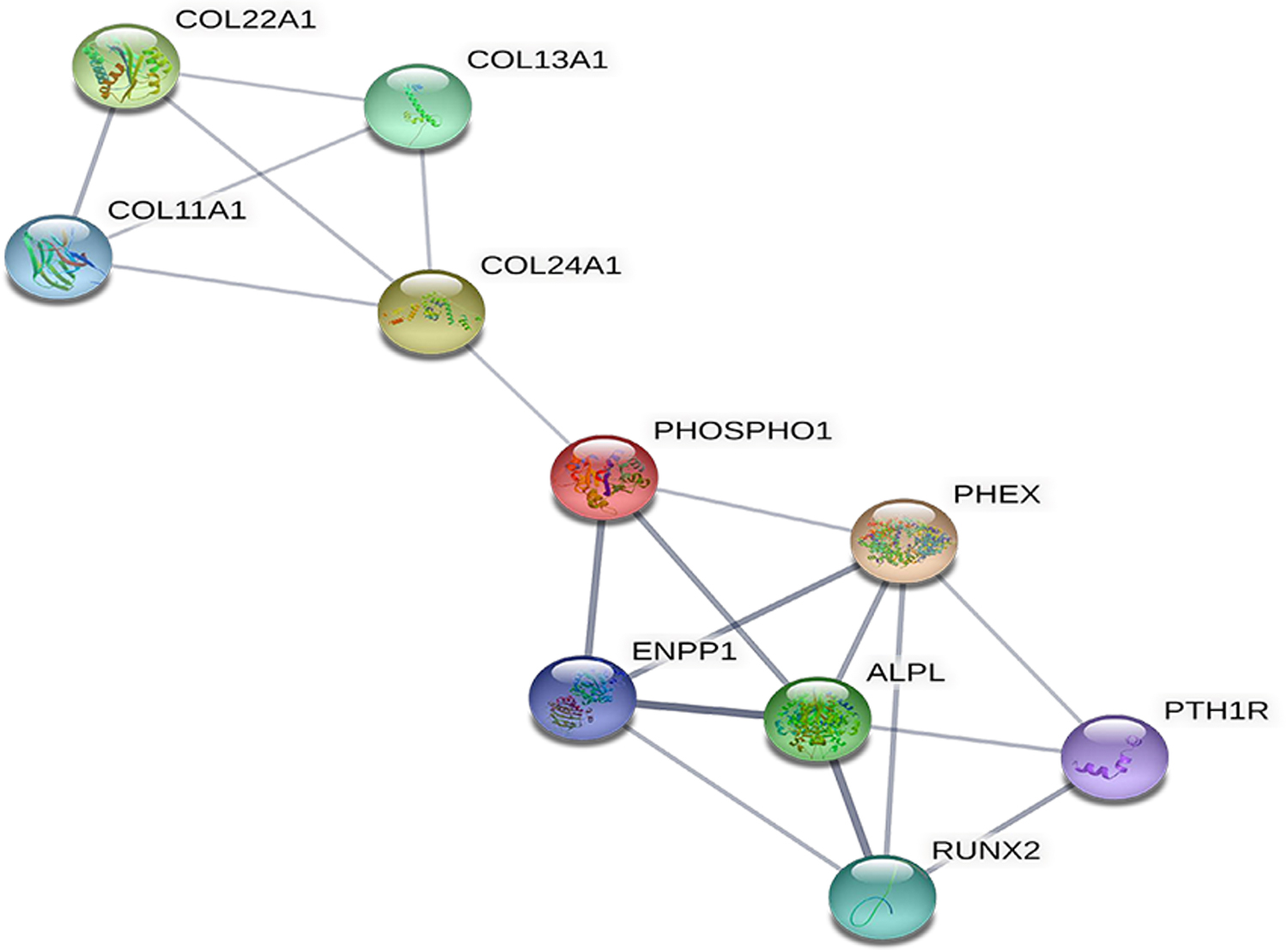
A Protein–protein interaction (PPI) network of statistically significant hub genes is identified, where the thickness of the edge (line) that connects associated nodes (circles) represents the strength of association between nodes. Note that node colors have no meaning and do not represent biological significance.

We constructed a protein–protein interaction (PPI) network for genes found in the cyan (M14) module (Figure 4). For visualization purposes, we constructed a gene co-expression network map based on the relationship and connectivity of genes using STRINGdb, a database consisting of known and predicted protein–protein interactions [17]. We chose genes with a score ≥0.4 to build a network model with 18 gene nodes (proteins) that we visualized with Cytoscape version 3.9.0 [18].

We selected candidate hub genes using the Cytoscape plugin called cytoHubba [19], which ranks nodes in the PPI network by their network features and scores each node gene by the top 10 algorithms: Maximal Clique Centrality (MCC), Density of Maximum Neighborhood Component (DMNC), Maximum Neighborhood Component (MNC), Degree, Edge Percolated Component (EPC), BottleNeck, EcCentricity, Closeness, Radiality, and Betweenness.

### 2.5. Statistical Analysis

To compare differential expression among tumor sample conditions, we performed statistical analyses using an unpaired two-tailed t-test in R Studio version 3.6.3 and considered *p* < 0.001 statistically significant.

### 2.6. Transcriptome Deconvolution and Tissue Expression Correlation Analysis

To estimate the proportion of immune and cancer cell make-up within the TIME from our bulk mCRPC RNA-Seq samples, we conducted bulk tissue transcriptome deconvolution analysis using a web-based tool called EPIC. A tab-deiminated text file displaying mCRPC samples with corresponding bulk gene expression counts given in fragments per kilobase per million mapped reads (FPKM) was used as the input into the tool (Table S1).

**Figure 5.**
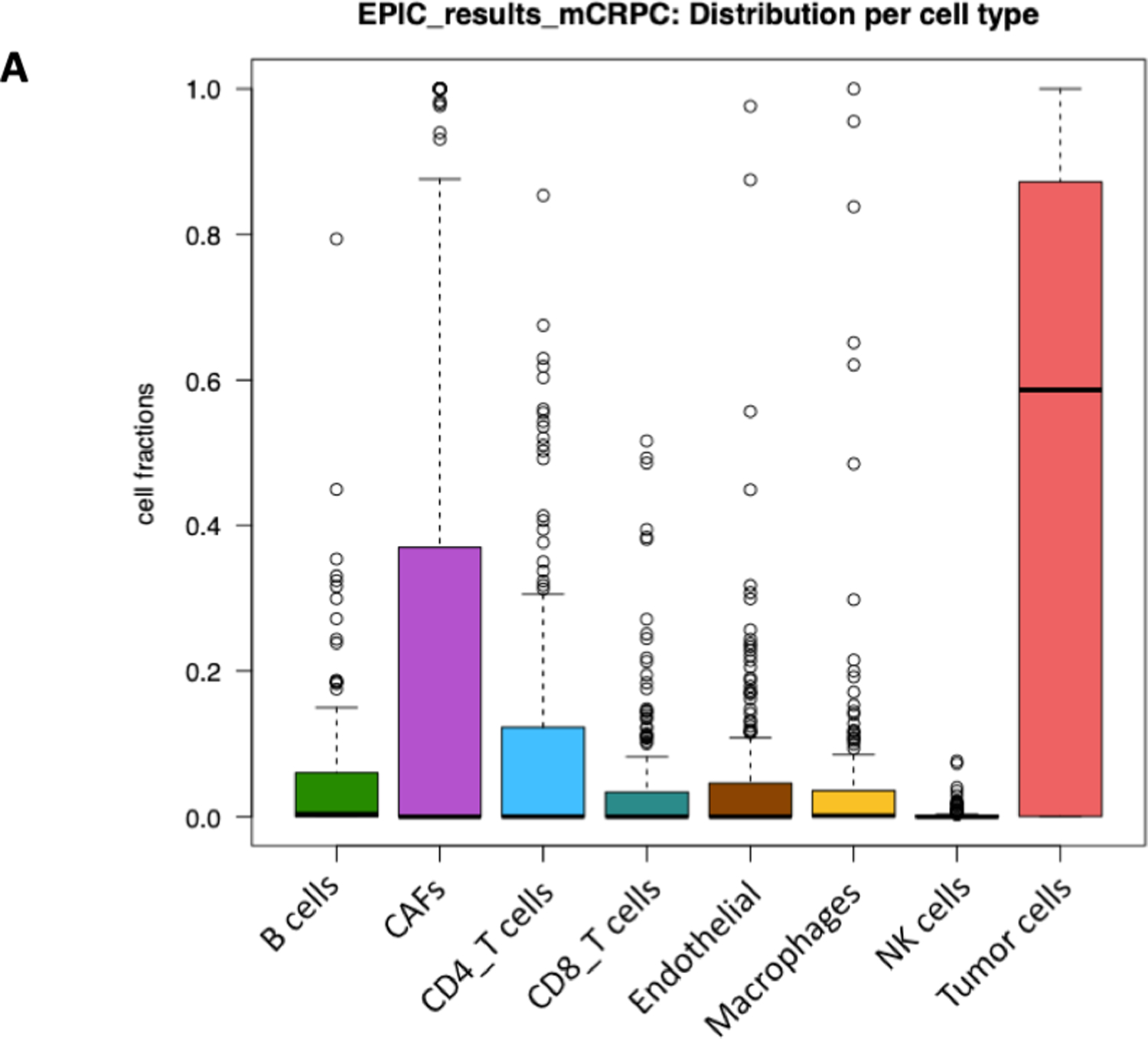

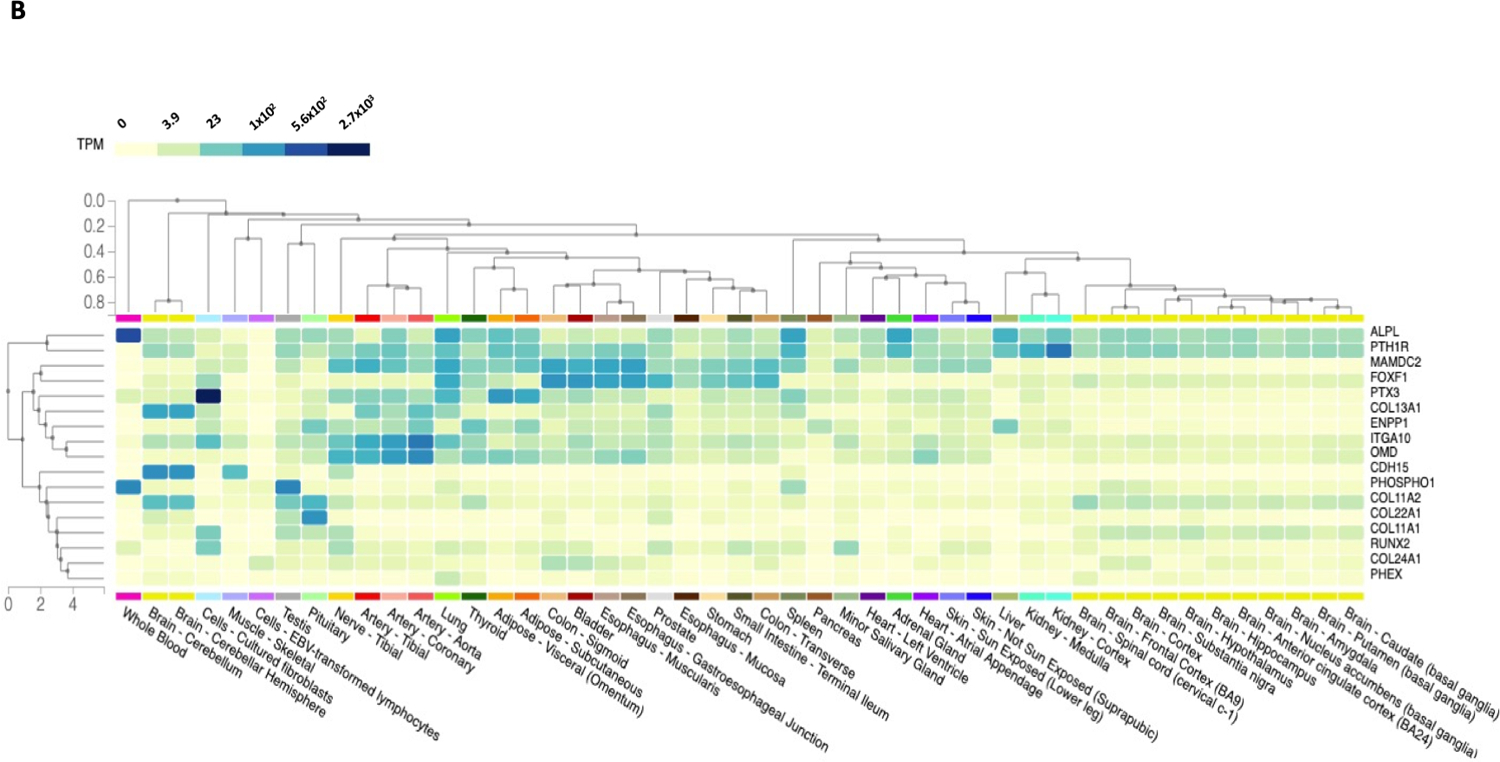
Distribution of tissue cell types and gene expression levels of hub genes across various tissue types. (**a**) The distribution of each cell type (B-cells, cancer-associated fibroblasts (CAFs), CD4 T-cells, CD8 T-cells, endothelial cells, macrophages, NK cells, and tumor cells) among mCRPC samples in the current dataset; (**b**) the hierarchical clustering of the diseases is shown as a dendrogram by column. The hierarchical clustering of the select differentially expressed genes is shown as a dendrogram by row. The center plot shows a correlation heat map of gene expression levels by genotype-tissue expression (GTEx) profiles. Gene and transcript expressions on the GTEx portal are shown in transcripts per million (TPM).

Finally, we employed GTEx, a web-based tool used to calculate the correlations between genotype and tissue-specific gene expression levels. We performed a multigene query using the genes most differentially expressed in our analysis. These genes included *ALPL*, *CDH15*, *COL11A1*, *COL11A2*, *COL13A1*, *COL22A1*, *COL24A1*, *ENPP1*, *FOXF1*, *ITGA10*, *MAMDC2*, *OMD*, *PHEX*, *PHOSPHO1*, *PTH1R*, *PTX3*, and *RUNX2*. GTEx did not have a bone tissue in their dataset for comparison, but upon comparison of our hub genes in prostate versus other tissue sites, we observed expression levels to be lower, on average, when compared to other tissue sites such as kidney, lung, whole blood, and spleen (Figure 5b).

## 3. Results

### 3.1. Identification of Co-Expressed Genes

We studied the transcriptomics of 60 mCRPC patient samples with matched clinical annotations. We chose the power of β = 9 (scale-free R^2^ = 0.90) to ensure scale independence for the scale-free network. We performed hierarchical clustering and Dynamic Tree Cutting to cluster co-expressed genes into modules (Figure 1).

We identified 14 modules from the construction of the eigengene network. These modules contained ≥30 genes per module: 5444 genes in the turquoise module (M1); 2174 genes in the blue module (M2); 1670 genes in the brown module (M3); 837 genes in the yellow module (M4); 556 genes in the green module (M5); 553 genes in the red module (M6); 385 genes in the black module (M7); 324 genes in the pink module (M8); 271 genes in the magenta module (M9); 125 genes in the purple module (M10); 82 genes in the green-yellow module (M11); 77 genes in the tan module (M12); 58 genes in the salmon module (M13); and 37 genes in the cyan module (M14).

### 3.2. Association of Modules with Clinical Traits

Figure 2 relates the clustered dendrogram to the matched clinical annotations, including age, tissue site, GS, taxane status, and hormone therapy status. The cyan module (M14) was most positively correlated with the presence of bone metastases (r = 0.81, *p* < 4 × 10^−15^). We found that other modules were positively correlated with the GS, such as the salmon module (M13; r = 0.37, *p* < 0.004), the tan module (M12; r = 0.34, *p* < 0.009), and the black module (M7, r = 0.4, *p* < 0.002).

### 3.3. Differential Gene Expression Analysis

We identified 3122 differentially expressed genes in the study dataset (Figure 3). We modeled our analysis to compare bone metastases with the other two metastatic sites, the lymph nodes and liver. We observed seven upregulated genes (*p* < 1 × 10^−10^) within the cyan module (M14) and one downregulated gene (FOXF1) in the yellow module (M11).

### 3.4. Enrichment Analysis of Biological Features

In the cyan module (M14), 23 of the 37 genes had >0.70 consensus kME values: *MAMDC2*, *PHOSPHO1*, *ITGA10*, *PTH1R*, *OMD*, *RUNX2*, *CTSK*, *CLMP*, *COL11A1*, *COL24A1*, *SHANK1*, *PTX3*, *CDH15*, *PLPP7*, *ALPL*, *COL22A1*, *MMP16*, *COL13A1*, *ADAMTS6*, *SATB2*, *ENPP2*, *EXTL1*, and *PHEX*. Two genes (*NMT1* and *PSMD3*) were negatively correlated with the presence of bone metastases and thus had low connectivity of membership to the cyan module (M14). We assessed comparisons with unpaired two-tailed t-tests and found *p* < 0.001 for all.

We used DAVID for GO and KEGG pathway analyses to afford a high-level view of the molecular signaling pathways that may drive gene co-expression in the cyan module (M14). We divided GO results into subdomains that represented biological processes, cellular components, and molecular functions, and we analyzed the top three GO terms for molecular functions with *p* < 0.01. The cyan module (M14) genes were mainly associated with endochondral ossification, skeletal system development, and collagen catabolic processes. KEGG enrichment analysis identified (*p* < 0.001) two signaling pathways involved in protein digestion and absorption and parathyroid hormone synthesis, secretion, and action.

### 3.5. Identification of Hub Genes

PPI network analysis provides protein-level context to biological processes and helps predict the functional interactions of key genes in pathogenic molecular processes. Our PPI network had an enrichment *p*-value of <1 × 10^−16^ and contained 18 nodes (proteins) with 31 edges. We used cytoHubba to rank the nodes by their network features. We generated a list of ranked candidate hub genes in the PPI network using Maximum Clique Centrality as a topological analysis method [19,20]. The top 10 candidate hub genes were *ALPL*, *PHEX*, *RUNX2*, *ENPP1*, *PHOSPHO1*, *COL24A1*, *PTH1R*, *COL12A1*, and *COL11A1* (Figure 4).

### 3.6. Transcriptome Deconvolution and Tissue Gene Expression Analysis

Results of our transcriptome deconvolution analysis showed a heterogenous mix of B-cells, cancer-associated fibroblasts (CAFs), T-cells, endothelial cells, macrophages, NK cells, and tumor cells (Table S2). Cell fractions per patient sample, on average, contained more than 60% tumor cells, followed by CAFs (~35%) (Figure 5a). GTEx analysis was performed to determine whether genes significantly differentially expressed in our *WGCNA* analysis were highly expressed in normal prostate tissue. Results show that our selected genes were not overexpressed in prostate tissue. However, ALPL was highly expressed in whole blood, PTX3 in cultured fibroblasts, and PHOSPHO1 in testis, spleen, and a few other tissue types.

## 4. Discussion

### 4.1. Key Findings in the Study

WGCNA holds great promise as a tool to interrogate human transcriptome data and elucidate molecular and signaling mechanisms for complex diseases, including PCa. In this study, we have characterized gene expression networks that may contribute to the heterogeneity and complexity of bone metastasis in mCRPC.

We identified 14 MEs positively or negatively correlated with age, GS, tumor site, hormone therapy status, and taxane exposure status. The cyan module (M14) was most positively correlated to the presence of bone metastases (R = 0.81, *p*-value = 4 × 10^−15^), and it contained 37 genes with potential clinical value. Enrichment analysis showed biological associations with absorption and reabsorption biological processes (e.g., endochondral ossification, replacement ossification, and endochondral bone morphogenesis) and signaling pathways involved in protein digestion and absorption parathyroid hormone synthesis, secretion, and action.

**Figure 6.**
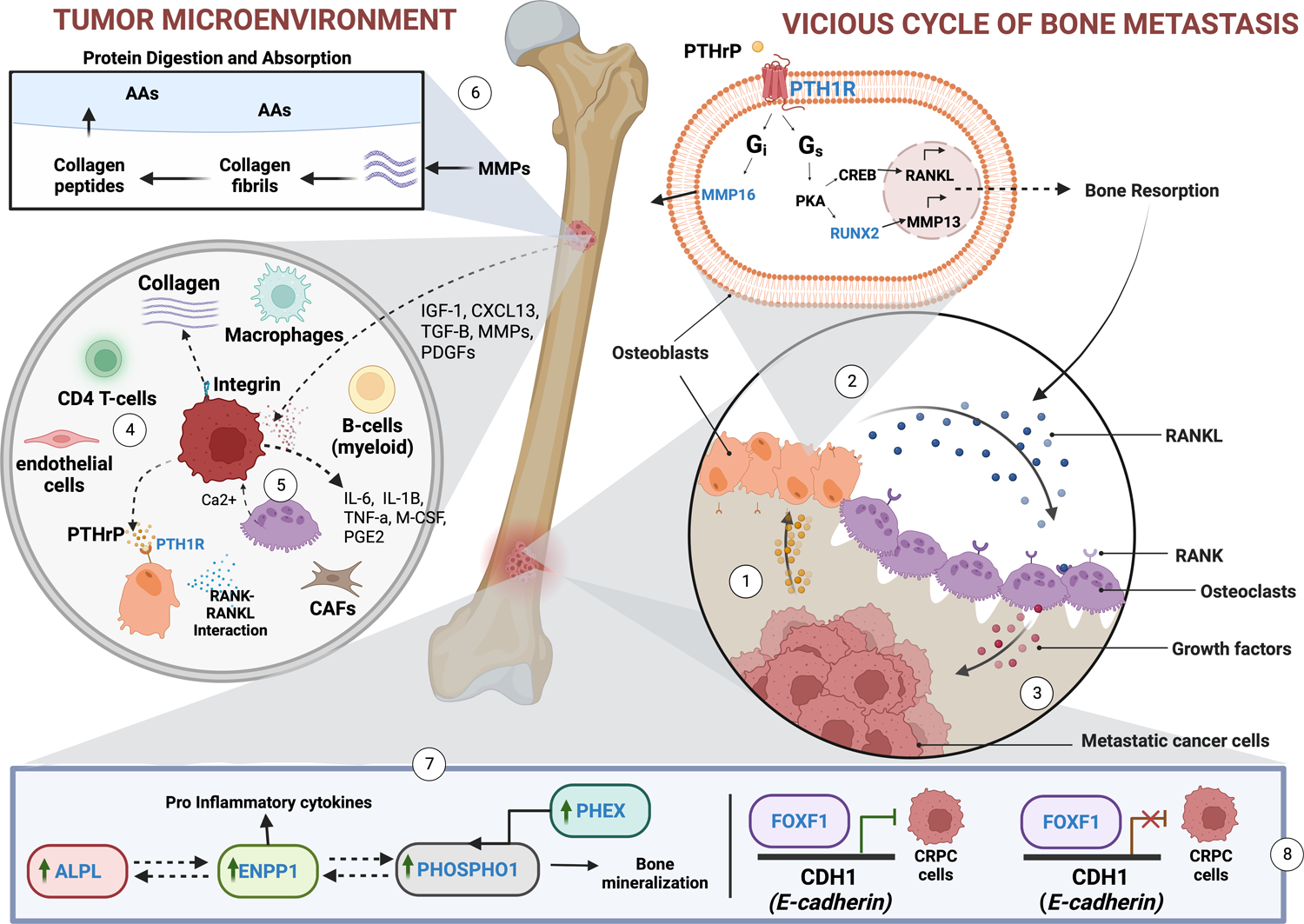
CRPC bone model of tumor microenvironment. Model displays the vicious cycle of bone metastasis driven by invading tumor cells. (1) PCa cells induce osteoblasts (OBs) to secrete RANKL. (2) RANKL binds to osteoclasts (OCs) and increases proliferation. (3) OCs promote increased resorption and pro-tumorigenic growth factors. (4) Tumor cells release PTHrP to reprogram OBs. (5) PCa cells release several other growth factors to promote OC proliferation and differentiation. Cancer-associated fibroblasts (CAFs), endothelial cells, myeloid B-cells, macrophages, T-cells, and other immune cell types interact with the tumor and the bone metastatic niche to promote tumorigenic activities. (6) PCa cells secrete factors that degrade the extracellular matrix, such as MMPs, composed mainly of collagen. Collagen may be reshaped, remodeled, or broken down into smaller peptides and proteins to be endocytosed via macrophages and fibroblasts to advance tumor growth, recycle amino acids, or be used for other functions that are favorable to the TIME. (7) PCa cells promote the overexpression of ALPL ENPP1, PHOSPHO1, and PHEX to create a favorable tumor microenvironment. (8) FOXF1 transcriptionally regulates cancer cell invasion and migration through the repression or silencing of the E-cadherin coding gene, CDH1. The downregulation or mutation of the FOXF1 gene increases the expression of E-cadherins, which promotes cancer cell invasion and motility. Created with BioRender.com.

Our analyses revealed 10 hub genes with statistical correlation to bone metastasis in patients with mCRPC: *ALPL*, *PHEX*, *RUNX2*, *ENPP1*, *PHOSPHO1*, *PTH1R*, *COL11A1*, *COL24A1*, *COL22A1*, and *COL13A1*. Two of these genes (*PHEX* and *PHOSPHO1*) have not been previously associated with mCRPC, while all other hub genes have some verified association with mCRPC or PCa [21–28]. Together, our analysis showed an interplay between the tumor microenvironment, the bone metastatic niche, and our hub genes. Results from both EPIC and GTEx show that the tumor leverages the overexpression of genes and associated genes to establish, maintain, and survive in an environment suitable for its growth and further metastasis. Here, we propose a model to show the vicious cycle of bone metastasis, driven, in part, by genes found significant in our study (Figure 6).

### 4.2. Hub Genes Not Previously Associated with mCRPC

The *PHEX* gene codes for an enzyme (phosphate regulating endopeptidase homolog X-linked) that helps regulate phosphate balance. PHEX has been hypothesized to regulate fibroblast growth factor-23 (FGF23), which inhibits 1,25 (OH)2D synthesis and may negatively regulate parathyroid hormone (PTH) secretion [29]. We are the first to report that *PHEX* overexpression may be involved with the dysregulated mineralization of skeletal tissue during mCRPC bone metastasis. We hypothesize that mCRPC cells may leverage the PHEX function to maintain the TIME and impede wound healing in bone.

PHOSPHO1 is well known in wound healing [30] but has not been described within the TIME for mCRPC in bone. Our study is the first to suggest that *PHOSPHO1* overexpression, enabled by *ALPL* and *ENPP1* crosstalk, may be involved in mCRPC bone metastasis through the dysregulated mineralization of skeletal tissue. *PHOSPHO1* is an attractive drug target, particularly for a series of benzoisothiazolinone inhibitors that have passed medicinal chemistry criteria and pose no cellular toxicity [31], but to our knowledge, there have been no therapeutic interventions with PHOSPHO1 inhibitors to date.

Hub genes associated with collagen (*COL11A1*, *COL24A1*, *COL22A1*, and *COL13A1*) and protein digestion and absorption signaling were upregulated in mCRPC samples with bone metastases. Cancer cells are known to reversibly reshape collagen to advance progression in a reinforcing cell–collagen loop [22], and our findings support and further resolve these molecular mechanisms. Further, research suggests that *COL11A1* upregulation is associated with decreased recurrence-free survival in PCa and could be targeted as a prognostic biomarker [32], but we could not expand these findings as survival data were not available in this study’s data set.

### 4.3. Hub Genes Previously Associated with mCRPC

The remaining hub genes (*ALPL*, *RUNX2*, *ENPP1*, and *PTH1R*) are involved in the overexpressed pathway of parathyroid hormone synthesis, secretion, and action signaling. A parathyroid hormone-related peptide, PTHrP, is believed to initiate bone resorption by upregulating RANKL and releasing other growth factors that promote the vicious cycle of bone metastasis into the bone TIME (Figure 6) [33]. The underlying mechanism remains poorly understood. Here, we outline the possible mechanisms involved in prostate cancer bone metastasis, driven, in part, by the hub genes.

#### 4.3.1. ALPL and RUNX2

ALPL plays a significant role in cell death and epithelial plasticity through its association with runt-related transcription factor 2 (*RUNX2*) and the receptor activator of nuclear factor kappa-b ligand (*RANKL*) signaling, both of which are downstream factors of parathyroid hormone signaling in the bone. Localized PCa cells express ALPL and significantly upregulate the *ALPL* gene for metastasis [24].

*RUNX2* and *RANKL* signaling promotes the tumorigenesis of mCRPC in bone [24,34,35]. *RUNX2* overexpression increases matrix metalloproteinase (*MMP*) expression and the invasion activity of the tumor, leading to PCa progression and metastasis [36,37]. The connective tissue growth factor (CTGF) reduces the ubiquitination-dependent degradation of *RUNX2* and promotes *RUNX2* acetylation in cancer cells, stabilizing *RUNX2* and thereby increasing the production of *RANKL* and MMPs. If left unchecked, *RUNX2* and *RANKL* signaling promotes osteoclasts to engage in a vicious cycle of bone matrix resorption and growth factor release that favors tumor growth and survival [38].

Taken together, our study further correlates *ALPL* and *RUNX2* signaling with the molecular signaling of *RANKL* and *MMP*, a modulation that affects the tumor cell invasiveness and phenotypic plasticity.

#### 4.3.2. ENPP1

Ectonucleotide pyrophosphatase/phosphodiesterase 1 (*ENPP1*) codes a transmembrane protein upregulated in many cancers, suppresses the innate immune response, and promotes tumor cell migration, proliferation, metastasis, and angiogenesis [26,39]. *ENPP1* inhibits pro-inflammatory cytokine production, stimulates anti-inflammatory cytokine synthesis, and leverages two GPCR receptors (A_2a_ A_2b_) to hydrolyze ATP, contributing to increased adenosine signaling in the hypoxic tumor microenvironment of metastatic PCa [40–42]. Our GO enrichment indicated that phosphodiesterase I activity is highly enriched in the cyan module, results that are supported by the *ENPP1*-to-adenosine signaling axis. Researchers are currently investigating the clinical utility of *ENPP1* inhibitors in many cancers [25].

#### 4.3.3. PTH1R

The parathyroid hormone 1 receptor (PTH1R) and calcium-sensing receptor (CaSR) create a favorable metastatic niche in bone through parathyroid hormone synthesis, secretion, and action. Yang and Wang showed that in breast cancer cells, which also metastasize to bone, CaSR activation upregulates the parathyroid hormone-related protein (PTHrP) and subsequently activates the G_s_/cAMP pathway that furthers PTHrP production in a “feed-forward” loop. Cancer cells release PTHrP, which binds to PTH1R in stromal cells or osteoblasts and causes RANKL production. RANKL then binds to the RANK receptor and spurs the maturation of osteoclasts, which reabsorb the bone matrix and release the calcium that binds to membrane-bound CaSR on tumor cells [28]. *PTHrP* ablation leads to a significant decrease in tumor growth and metastasis as well as the reduced expression of several factors known to support tumor progression, including: CXCR4, Ki67, Bcl-2, AKT1, and Cyclin D1 [27]. Our identification of *PTH1R* as a hub gene further supports its role in forming a tumor microenvironment in bone.

### 4.4. FOXF1

The forkhead box protein F1 (*FOXF1*) gene codes for a protein that is thought to transactivate *CDH1* and upregulate the expression of the associated membrane protein, Ecadherin. When combined with a mutated or deleted p53 gene, common in PCa progression cases, downregulated *FOXF1* may reduce E-cadherin expression and promote metastasis by creating a survival advantage for motile and invasive tumor cells [6]. Our analysis observed *FOXF1* downregulation when *RUNX2* was upregulated in patients with mCRPC in bone.

### 4.5. Study Limitations

This study generates novel insights into the biological pathways associated with bone metastasis in mCRPC. However, we used bioinformatic analysis for our study, so future research is required to validate the role of hub genes in tumorigenesis and progression. Furthermore, our analysis was not able to determine the direction of signaling effects. Our characterization of signaling and pathways differences was solely assessed based on current literature, gene ontology, and protein–protein interaction analysis. Lastly, although the validation and replication of these discoveries are important processes that serve to support the conclusions made in the current study, the datasets needed for the analysis of metastatic prostate tissue to bone are currently not available in a sample size large enough for robust comparison.

## 5. Conclusions

We present a novel and comprehensive systems biology approach to further our understanding of the molecular and biological mechanisms involved in the TIME niche for mCRPC. We used WGCNA to construct a network and identified 14 modules, and the cyan module (M14) was enriched with genes that were positively correlated to bone metastasis. We discovered two novel hub genes that warrant further investigation for their molecular role in mCRPC, especially as candidate hub genes may serve as molecular targets or diagnostic biomarkers for precise diagnosis or cancer treatment.

## Supplementary Material

Table S1

Table S2

## Figures and Tables

**Table 1.** Clinical data for mCPRC patient samples

Clinical variable		Total number
Total		60
Age	Median [range]	65 [50 – 85]
	UNK	8
Tissue Site		
	Bone	15
	Lymph Node	34
	Liver	11
Abiraterone and Enzalutamide Exposure (Hormone Therapy) Status		
	Naïve	31
	Exposed	25
	UNK	4
Taxane Exposure Status		
	Naïve	37
	Exposed	21
	UNK	2
Gleason Score		
	6	3
	7	9
	8	9
	9	19
	10	10
	UNK	24

Five clinical traits serve as the variables for this study, as shown in the first column. They include age, tissue site, hormone therapy status, taxane exposure status, and Gleason score. In the second column, taxane exposure and hormone therapy status were recoded as binary (0 and 1, Naïve and Exposed, respectively). Gleason score was coded as discrete values, and age was coded as continuous values. Tissue site was segmented to be binary (0 and 1). For example, bone = 0 for non-bone and 1 for bone. UNK = unknown. A total of 60 patient samples were used in this study, as shown in the third top column.

**Table 2.** Signaling pathways from KEGG pathway analysis, including pathway names, gene names, percentage of all genes (n=37), p-values, and Benjamini-Hochberg procedure values.

Pathway	Genes	%	p-value	Benjamini-Hochberg Value
Protein digestion and absorption	***COL11A1***	10.8	2.2E-3	1.3E-1
	***COL13A1***			
	***COL22A1***			
	***COL24A1***			
Parathyroid hormone synthesis, secretion, and action	***RUNX2***	8.1	8.6E-1	8.6E-1
	***MMP16***			
	***PTH1R***			

## Data Availability

The data presented in this study are openly available in Cbioportal [6,43], GitHub [7], and dbGaP [44].
